# Anesthetic management of folders with severe kyphosis in ankylosing spondylitis: a single-center retrospective case series study

**DOI:** 10.3389/fmed.2025.1503912

**Published:** 2025-04-16

**Authors:** Lin Peng, Qiang Li, Lingxi Zheng, Deng Zhao, Qiang Fu

**Affiliations:** ^1^Department of Anesthesiology, The Third People’s Hospital of Chengdu (The Affiliated Hospital of Southwest Jiaotong University), College of Medicine, Southwest Jiaotong University, Chengdu, Sichuan, China; ^2^Department of Anesthesiology, The Third People’s Hospital of Chengdu, Southwest Jiao Tong University, Chengdu, China; ^3^Department of Orthopaedics, The Third People’s Hospital of Chengdu, Southwest Jiao Tong University, Chengdu, China

**Keywords:** anesthetic management, ankylosing spondylitis, kyphosis surgery, severe kyphosis, hemodynamic monitoring

## Abstract

**Background:**

Ankylosing spondylitis (AS) is a progressive inflammatory disease causing severe kyphosis, which complicates surgical management and increases complication risks. This study aims to analyze the characteristics of severe kyphosis in AS and explore methods to optimize perioperative management and reduce complications.

**Methods:**

We conducted a retrospective analysis of clinical data from five patients with severe kyphosis in AS who underwent surgery between October 2017 and February 2022. The patients had a mean age of 40.20 ± 8.50 years. The analysis included pathophysiological changes in folded patients and perioperative multidisciplinary intervention guidance. It also covered strict preoperative anesthetic evaluations, establishing an optimal fluid pathway during surgery, precise anesthetic monitoring and management, and applying postoperative multimodal analgesia and rehabilitation exercises to optimize perioperative anesthetic management.

**Results:**

Preoperative cardiopulmonary function exercises were required to ensure patients could withstand surgery and anesthesia. Awake fiberoptic tracheal intubation was used to ensure airway safety and anesthesia. Hemodynamic evaluation and management were conducted using PICCO monitoring. Somatosensory evoked potentials (SSEP) and myogenic motor evoked potentials (MMEP) were utilized for neural axis monitoring. Hypothermia was designed to protect the spinal cord. To prevent massive blood loss, controlled hypotension and autotransfusion were implemented.

**Conclusion:**

The correction operation of severe spinal kyphosis is complex and requires a detailed anesthesia plan. Optimizing the management of difficult airways and respiratory regulation, guiding circulation and fluid management through comprehensive monitoring, avoiding factors that aggravate complications, improving postoperative analgesia, and encouraging active rehabilitation exercises are crucial goals for perioperative anesthesia management.

## 1 Introduction

Ankylosing spondylitis (AS) is a progressive chronic inflammatory disease, primarily affecting the spine and sacroiliac joints and causing fusion and rigidity of the spine (‘bamboo spine’), and eventually developing into cervical rigidity and thoracic and lumbar kyphosis (1). AS is the most common cause of kyphotic deformity, with an incidence rate of approximately 49%, and it affects approximately ~0.1–1.4% of the population (2–4). Kyphosis results from abnormal backward protrusion of the spine, altering the anatomical shape of the spine and its associated tissues. Severe kyphosis often prevents patients from walking upright and looking straight ahead due to disrupted gravity balance, most affected individuals are thin and short in stature (5). Spinal deformity, particularly kyphosis, can lead to restrictive respiratory dysfunction due to thoracic deformity, impacting exercise tolerance. A significant reduction in these volumes of the thoracic and abdominal affects the normal activity space of the heart in the mediastinum, resulting in a smaller cardiac cavity and greatly impacting left ventricular diastolic function (6, 7). Anesthesia and kyphosis surgery are associated with significantly increased morbidity and mortality due to complications such as right ventricular failure, arrhythmia, postoperative hypoxemia and myocardial ischemia (8). The pathophysiology of severe kyphosis, screening for surgical risk factors, preoperative evaluation, comprehensive multidisciplinary planning, intraoperative monitoring and management, and prevention and treatment of postoperative complications pose significant challenges for anesthesiologists.

Therefore, we present successful cases of primary surgery for severe kyphosis in ankylosing spondylitis. This study aims to provide a reference for perioperative anesthesia management and treatment of such cases.

## 2 Methods

### 2.1 Patient selection

This single-center, retrospective study was granted by the institutional review board of the hospital, with the requirement for written informed consent waived by the ethics committee, all patient data utilized in this study were handled in strict adherence to ethical guidelines. Inclusion criteria were confirmed AS patients with kyphosis as the main deformity, presenting in a folded state (measured Cobb’s angle ≥150°), and complete clinical and auxiliary examination data. Exclusion criteria were primary thoracic deformities, congenital heart disease, history of cardiothoracic surgery, history of spinal trauma or spinal surgery, and inability to undergo the necessary monitoring program (including Psychological or physical limitations; Severe physical illnesses; Inability to undergo specific tests; Inability to follow the follow-up plan; Inability to provide necessary information; Legal or ethical constraints, etc.). A retrospective analysis was conducted on AS patients with severe kyphosis who underwent surgery at the hospital from October 2017 to February 2022. Five patients meeting these criteria were selected.

### 2.2 Data collection

Demographic data such as age, height, weight, and arm length, as well as comorbidities and New York Heart Association (NYHA) cardiac function status before, during, and after kyphosis surgery were collected. Additional data included results from the 6-min walking test, breath-hold test, and other existing comorbidities. Preoperative examinations included pulmonary function tests, cardiac color ultrasound, electrocardiogram, routine blood tests, arterial blood gas analysis, liver and kidney function tests, blood coagulation tests, and neuro electrophysiology. All patients underwent spinal X-ray examination standing, with Cobb’s angle and global kyphosis angle (GK) measured on anteroposterior and lateral films using Surgimap software.

### 2.3 Statistical analysis

Statistical analysis was performed using SPSS 25.0 software. Measurement data are expressed as mean ± standard deviation (x ± S). A *p*-value of <0.05 was considered statistically significant.

## 3 Results

### 3.1 Basic demographic characteristics of patients with kyphosis underwent surgery in ankylosing spondylitis

All patients with AS were confirmed. Table 1 presents their baseline information and management before kyphosis surgery. Once hemodynamics stabilized post-extubation, all patients were transferred to the general ward for additional treatment.

**Table 1:** Demographic characteristics and clinical features.

Parameter	Value					Normal ranges
Age (years)	38	52	30	36	45	—
Weight (kg)	46	41	44	35	38	—
Cobb’s angle (°)	152.07	150.97	151.89	153.15	151.62	—
GK angle (°)	129	121	140	151	140	—
Height (cm)	85	86	97	66	73	—
Arm length (cm)	169	168	159	175	165	—
HR (beats/min)	100	111	92	88	80	60–100
BP (mmHg)	130/78	115/95	126/82	97/64	105/56	90–120/60–80
SpO_2_ (%)	97.9	98.6	98.7	96.7	97.4	95–100
Na^+^ (mmol/L)	138.8	138.4	141.1	140.8	138.0	135–145
K^+^ (mmol/L)	4.2	4.18	4.25	4.04	4.66	3.5–5.0
(BUN) (mmol/L)	6.89	5.05	3.40	4.69	8.48	2.5–7.5
Creatinine (μmol/L)	51.5	52.7	53.8	71.7	77.5	53–106
Hb (g/L)	113	115	113	106	123	120–150
WBC (×10^9^/L)	6.12	5.06	6.64	7.79	11.39	4.0–10.0
PLT (×10^9^/L)	132	428	79	250	409	125–350
Pulmonary function	Moderate	Severe	Moderately severe	Very severe	Severe	—
Joint involvement	Hip	Hip	No	Hip & knee	Hip & knee	—

**Figure 1:**
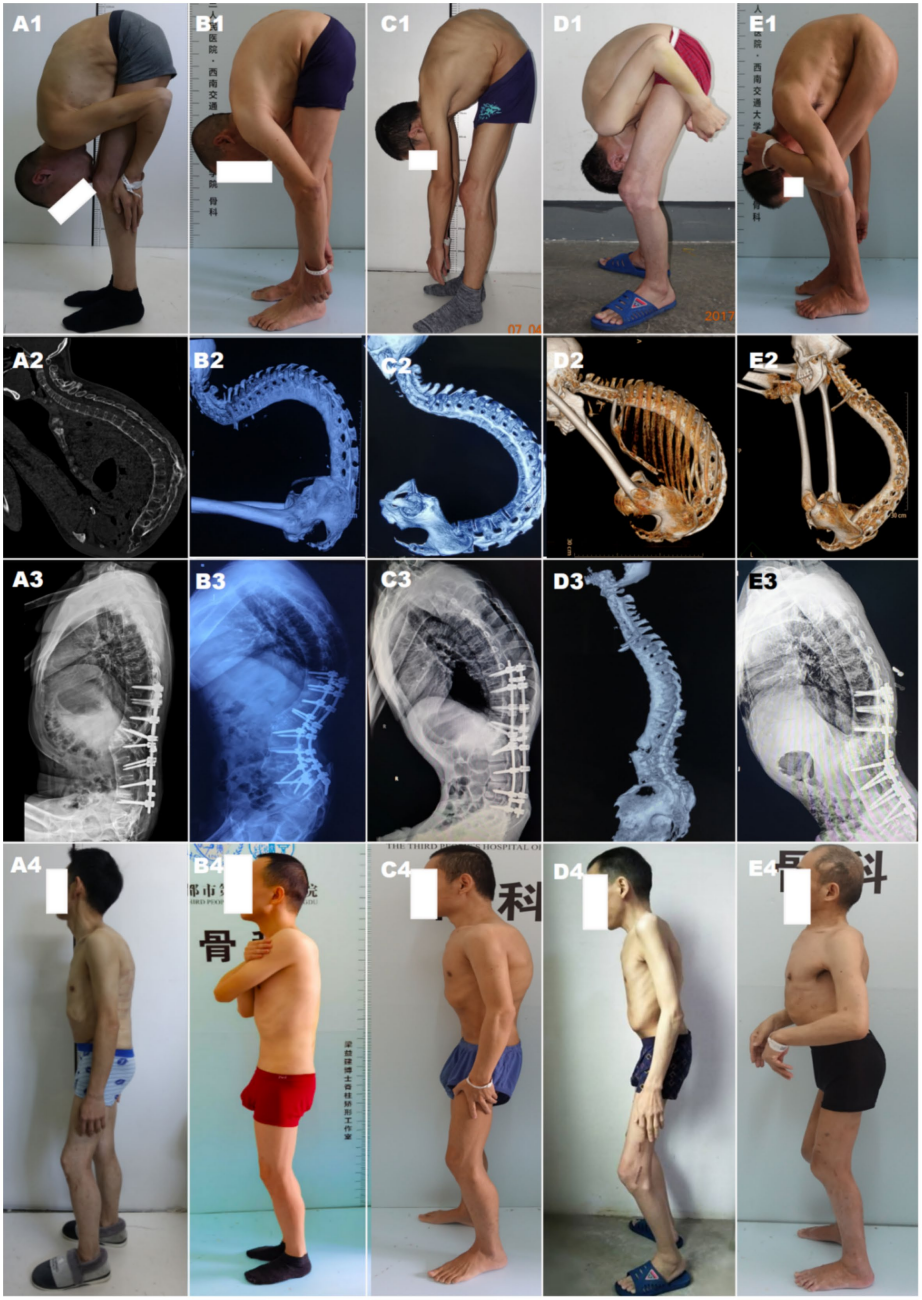
Pre-and postoperative presentation and changes in radiographic spinal level maps. **(A–E)** represent five patients. **(A1–E1)** Preoperative standing posture showing thoracolumbar kyphotic deformity; **(A2–E2)** Preoperative 3D CT reconstruction of the spine; **(A3–E3)** Postoperative spine alignment shown on X-ray/CT; **(A4–E4)** Postoperative standing posture changes.

During the first stage, patients underwent a single level Ponte osteotomy (SPO) in the lateral position. The correction result of the first-stage surgery was assessed utilizing Cobb’s angle, with positive values indicating kyphosis (Figure 1).

### 3.2 Preoperative evaluation and management

The preoperative preparation time ranged from 2 weeks to 6 months. Vital capacity was improved through airbag blowing, lip contraction and exhalation exercises, and stair climbing, enhanced cardiopulmonary function. Nutritional enhancement was provided to emaciated patients, and those with reflux esophagitis were treated with proton pump inhibitors (PPIs). All five patients showed obvious lung function impairment during exercise compared to the same age group. Before the operation, one patient (20%) had extremely severe ventilatory dysfunction, with an FVC value less than 40% of the reference value. Three patients (60%) had varying degrees of restrictive ventilatory dysfunction. CT scans revealed ankylosing spondylitis involvement in bilateral hip joints in four patients (80%) and bilateral knee joint involvement in two patients (50%). One patient (20%) had a history of hypertension.

An anesthesiologist evaluated the patients one day before the operation, focusing on several key points (more detailed assessments are shown in Table 2): All patients’ cervical vertebrae were fixed in the anterior flexion position, with limited extension, lateral flexion and rotation; the mouth opening of the patient was slightly limited, with no significant abnormality in the thyromental distance; there’s no severe airway compression or chest deformity was observed. Normal activity in both upper limbs, limited movement in both lower limbs, and a preference for sleeping in the lateral position; patients had difficulty walking upright, and the amount of activities was small. According to ASA clinical practice guidelines (9), three patients were classified as grade III, and two as grade IV, with more severe lung function impairment. Patients needed central venipuncture before the operation, one delayed the operation due to difficulty exposing the bilateral femoral and right internal jugular veins, but the second puncture was completed successfully after one week. The mental state evaluation and psychological counseling of the patient. The diagnosis and treatment plans were discussed preoperatively in a multidisciplinary meeting.

**Table 2:** Assessment index of difficult airway.

Evaluation indicators	Reference	Method & definition	Test grading	Judgment criteria
Modified Mallampati test	Wang LY (49), 2022	Quote: “Patient was asked to sit up with his mouth open as much as possible and to stick out his tongue without making a sound.”	Class I: The pharyngeal arch, soft palate and uvula Class II: The pharyngeal arch, soft palate and uvula, but the uvula is blocked by the tongue Class III: Soft palate and base of uvula Class IV: Hard palate only	III & IV are considered predictors of difficult intubation.
Wilson risk score	Roth D (14), 2018	Risk factor criteria score Weight: <90 kg (score = 0), 90 kg to 110 kg (score = 1), >110 kg (score = 2) Head and neck movement: >90° (score = 0), 90° (i.e., ±10°) (score = 1), <90° (score = 2) Jaw movement: IG ≥ 5 cm (score = 0), IG < 5 cm (score = 1), IG < 5 cm (score = 2) Receding mandible: normal (score = 0), moderate (score = 1), severe (score = 2) Buck teeth: normal (score = 0), moderate (score = 1), severe (score = 2)	Quote: “The maximum possible score is 10. Higher scores are considered to be predictive of a DA. The chosen cut-off points have been >2 or >4.”	>2
Thyromental distance	Baker PA (50), 2009	Distance of the nail cartilage notch to the tip of the lower jaw during the head extension position	Grade 1: >6.5 cm implies easy laryngoscopy and intubation; Grade 2: 6.0 cm to 6.5 cm implies difficult intubation but possible; Grade 3: <6 cm implies that intubation may not be possible.	Grade 3: <6 cm
Mouth opening test	Agrawal J (51), 2015	Spacing between the upper and lower incisors during the maximum mouth opening	Shorter distances are considered to be predictive of a DA.	<3 cm or 2 horizontal fingers
Upper lip bite test	Faramarzi E (52), 2018	The patient extends the lower incisor beyond the upper incisor	Class I. Lower incisors bite the upper lip above the vermilion border, mucosa not being visible Class II. Lower incisors bite the upper lip below the vermilion border, mucosa partially visible Class III. Lower incisors fail to bite the upper lip	Class III
Atlanto-occipital movement	Dhwani N Trambadia (15), 2023	The patient’s head is bent forward and down to contact the sternum, and then the face is raised to test the neck extension range	Estimated grading: Grade I: no decrease in atlanto-occipital joint extension; Grade II: 1/3 decrease; Grade III: 2/3 decrease	The chin must not touch the sternum or extend the neck

DA, difficult airway; IG, interincisor gap.

**Figure 2:**
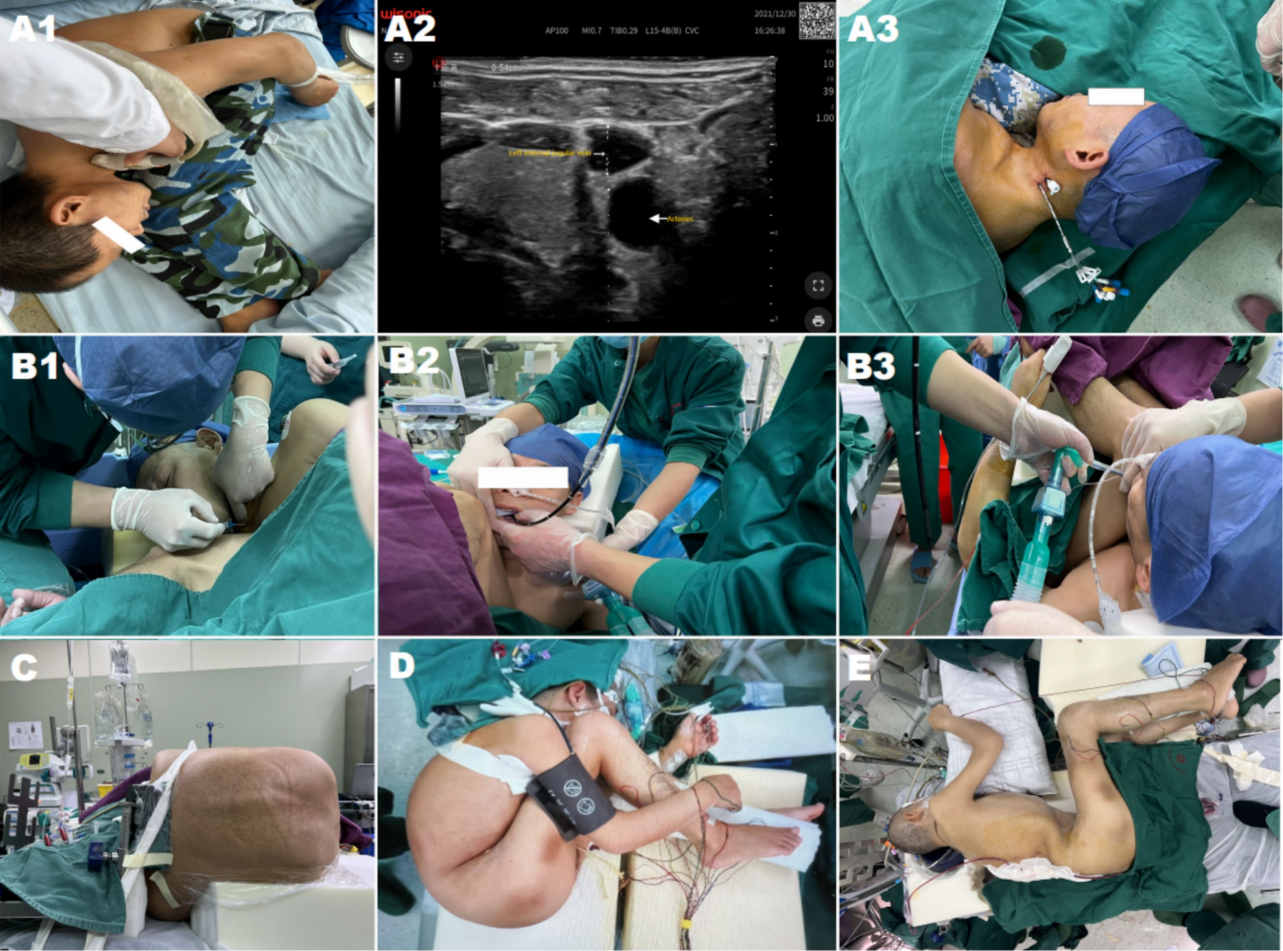
**(A1–A3)** Display the ultrasonic evaluation of blood vessels and establishment of a central vein. **(B1–B3)** Exhibit awake endotracheal intubation was performed after ultrasound-guided cricothyroid membrane puncture and tracheal mucosal anesthesia before endotracheal intubation. **(C)** Shows surgery was performed in a lateral position. **(D)** Depicts basic intraoperative vital signs and hemodynamic monitoring. **(E)** Expose the patient corrected the posture after the first-stage surgery.

As shown in Figure 2, a typical 45-year-old “folding patient” with severe kyphosis and hip ankylosis was reported. T12–L2 pedicle subtraction osteotomy (PSO) was performed in one stage, along with the anesthetic details, monitoring, patient status.

### 3.3 Intraoperative details of anesthesia monitoring and management in kyphosis surgery

Routine monitoring of heart rate (HR), non-invasive blood pressure (NIBP), SpO_2_ and body temperature was conducted upon entering the operating room. An internal jugular vein catheter was placed on the unobstructed side of the patient under ultrasound guidance before anesthesia, allowing for central venous pressure (CVP) monitoring during the operation. Due to the excessive kyphosis, the surgery was performed in the lateral position. During awake tracheal intubation, the patient was instructed to take deep breaths while being preoxygenated with 100% oxygen to increase the end-expiratory oxygen concentration to over 90%, thereby preventing an increase in arterial carbon dioxide concentration and ensuring sufficient oxygenation. This approach provided valuable time for anesthesiologists to perform the intubation. For anesthesia induction, intravenous injections of penehyclidine hydrochloride 0.3 mg, methylprednisolone 40 mg, and oxycodone 3 mg were administered. Simultaneously, dexmedetomidine was pump-infused at a loading dose of 0.5 μg/kg and a maintenance dose of 0.2 μg/kg/h. Blood pressure was monitored via radial artery catheterization, and pulse contour continuous cardiac output (PICCO) monitoring was used for all five patients. After topical anesthesia of the oropharynx with 1% tetracaine and cricothyroid membrane puncture, awake tracheal intubation was performed under fiberoptic bronchoscope guidance. The respiratory rate (RR) was maintained at 18–22 breaths per minute, with a fresh air flow of 2 L/min and a FiO_2_ of 50%, maintaining the PETCO_2_ between 35 and 45 mmHg. A lung protective ventilation strategy was employed with a tidal volume (VT) of 5–8 mL/kg, PEEP of 5 cmH_2_O, periodic lung reexpansion, lung expansion at an interval of 2 h, and lung expansion pressure of 30 cmH_2_O for 30 s. Ventilation mode and parameters were adjusted based on changes in airway pressure and dynamic lung compliance (Cdyn).

Anesthesia was maintained by a target-controlled infusion of total intravenous anesthesia (TIVA) of propofol, remifentanil and a maintenance dose of dextromethomide. The BIS values were kept between 45 and 60. Intraoperative targeted fluid management, autologous blood transfusion, and intermittent blood sampling were implemented for blood gas analysis. Fluid therapy and vasoactive drug administration were guided by intraoperative blood loss, blood gas indices, arterial pressure (AP), central venous pressure (CVP), pulse pressure variability (PPV) index, systemic vascular resistance index (SVR), and cardiac output measured by PICCO. Four patients developed hypovolemic hypotension (systemic blood pressure < 90/60 mmHg) during the operation and were treated with norepinephrine or epinephrine to maintain hemodynamic stability. The crystalloid to colloid infusion ratio was controlled at 2:1, and red blood cells were supplemented when hemoglobin (Hb) was lower than 90 g/L. Patients were kept warm with a heated blanket, maintaining bladder temperature between 36.0 and 37.0°C.

Continuous monitoring of somatosensory evoked potentials (SSEP) and myogenic motor evoked potentials (MMEP) should be performed in the absence of muscle relaxants and under Total Intravenous Anesthesia to assess for any injury to the neural axis. When the osteotomy was fixed with Lugo’s rod, an intraoperative awakening was required to check for spinal cord injury or compression. Propofol and remifentanil were stopped, and only dexmedetomidine was continued intravenously. Patients were awakened when BIS values exceeded 75, and they accurately moved their lower limbs as instructed, with an awakening time of approximately 15 min. At the end of the operation, four patients returned to spontaneous breathing, with measured tidal volumes reaching the predicted values and their consciousness and muscle tension returning to normal. Patients were monitored for 15 min after extubation before being transferred to the orthopedic ward. One patient required delayed extubation and subsequent transfer to the ICU for further management due to the recovery of only consciousness without adequate respiratory muscle strength. This patient was successfully extubated after achieving adequate tidal volume 17 h later and was transferred back to the orthopedic ward after 2 day of treatment in the ICU.

### 3.4 Postoperative management and rehabilitation

Postoperative analgesic therapy was administered to all patients, including oral NSAIDs, intravenous analgesics, and analgesic pumps. The postoperative patient-controlled intravenous analgesia (PCIA) regimen included oxycodone 50 mg, tropisetron 10 mg, and 100 mL of normal saline. The PCIA settings were as follows: no background dose, single injection dose of 4 mL, lockout time of 5 min, and a maximum dose of 20 mL in 4 h. None of the patients experienced operation-related neurological impairment. Four patients were successfully extubated while awake. One patient required ventilation with CPAP and SIMV modes in the ICU and was extubated 17 h postoperatively. The patient with delayed extubation continued non-invasive positive pressure ventilation for one day, with no need for a tracheotomy. All patients were required to undergo rehabilitation training to reduce the risk of postoperative complications, improve cardiopulmonary function and physical fitness, strengthen nutritional intake, and prepare for the second-stage operation.

### 3.5 Postoperative complications

There’s no neurological deficits, vascular injuries or other severe complications were observed. More surgery and anesthesia-related complications are shown in Table 3.

**Table 3:** Surgery and anesthesia-related complications.

Complication	Frequency (*n* = 5)	Outcome
Pneumonia	1	Cured
Atelectasis	1	Recovered
Pleural effusion	1	Cured
Surgical site infection	3	Cured
Urinary tract infection	2	Cured
Postoperative pain	1	Recovered
Gastrointestinal dysfunction	1	Healed
Pressure injury	1	Healed
Incomplete paraplegia of lower limbs	1	Resolution

## 4 Discussion

The underlying condition of rigid kyphosis (hunchback) in AS is characterized by extensive heterotopic bone formation in inflamed spinal ligaments, the increased incidence of compression fractures caused by AS osteoporosis further aggravates this problem (1). Kyphosis causes the center of gravity in the sagittal plane to move forward and backward. When ankylosis occurs, only the movable lower limb joints can compensate for the shift in the center of gravity, and stability is maintained by knee flexion and ankle base flexion (10). In the first stage of surgery, all patients underwent pedicle osteotomy and orthopedic bone graft fusion. Orthopedic osteotomy is considered an effective method for treating kyphosis in AS (11).

Strict preoperative evaluation before surgery is necessary. Patients with severe kyphosis often present a predictably difficult airway. A difficult airway event involves one or more sequential steps in upper airway management that are challenging or impossible to complete (12). There is no reference standard for a difficult airway, but it encompasses difficult mask ventilation, difficult laryngoscopy, difficult supraglottic airway ventilation, difficult or failed endotracheal intubation and extubation, failed airway establishment, and inadequate ventilation according to the 2022 American Society of Anesthesiologists Practice Guideline for Management of the Difficult Airway (13). Simple and direct assessment of patients with foreseeable, difficult airways is important in reducing adverse anesthetic events due to airway failure. The implementation of the modified Mallampati test, Wilson risk score, thyromental distance measurement, mouth opening test, upper lip bite test, and atlanto-occipital movement assessment by the anesthesiologist helps to understand the patient’s airway condition before surgery and prepare for unexpected intubation failure (14, 15).

Ultrasound, as a new airway assessment tool, is a better predictor of difficult airway. Ultrasound-based measurements can help assess airway compression or deformity (16, 17). Due to their severe deformities, these patients often cannot undergo conventional video laryngoscopy intubation or mask ventilation. Therefore, optimizing the patient’s oxygenation, airway management, and tracheal intubation is crucial, with particular emphasis on preoxygenation and apneic oxygenation. In such cases, performing a cricothyrotomy in the awake state before anesthesia induction is recommended as a reliable backup for failed tracheal intubation (18).

Fiberoptic bronchoscope conscious endotracheal intubation (FBI) is a feasible option for these patients. Awake supraglottic airway-guided FBI (SAGFBI) offers advantages over conventional awake FBI or awake video laryngoscopy. It reduces patient intolerance and associated complications and provides the benefits of “awake test insertion” of the supraglottic airway device (SAD), “awake observation” of the periglottic area, and “awake test ventilation” (19). Research has shown that for patients who cannot lie supine, the face-to-face intubation technique is an effective alternative (20). This technique takes less time in patients with predicted respiratory difficulties than video laryngoscopy. It also causes less damage to the pharyngeal wall or soft tissue and is less likely to result in intubation trauma since it enters along the base of the tongue (21). Compared with a direct laryngoscope, optical intubation tends to cause less hemodynamic instability (22).

Imagama et al. (23) showed for the first time that kyphosis, sagittal imbalance, increased daily oral medication and decreased back muscle strength are important risk factors for the development of GERD symptoms. In patients with a stiff spine and Cobb’s angle greater than 150°, preoperative multidisciplinary consultation considers gastroesophageal reflux caused by increased intra-abdominal pressure due to long-term compression of abdominal organs. Therefore, PPIs are administered to treat gastroesophageal reflux disease during the perioperative period. According to the Enhanced Recovery After Surgery (ERAS) program in Tong Y’s study (24), the digestive organs of these patients have been chronically compressed, leading to difficulties in eating and reduced nutritional intake, which in turn has caused cachexia. Therefore, nutritional support was provided during the perioperative period to optimize their preoperative and postoperative conditions.

Previous studies have consistently supported the beneficial effects of exercise on AS, backed by substantial mixed-quality evidence (25). Considering the moderate-to-severe restrictive ventilatory dysfunction and limited lower limb mobility, the preoperative exercise regimen included increasing the duration of flat-ground walking, balloon blowing exercises, and stair climbing. Preoperative pulmonary function tests revealed one patient with very severe respiratory dysfunction and four with moderate-to-severe restrictive ventilatory impairment, with no reports of pulmonary hypertension. These cardiopulmonary abnormalities increased the risk of anesthesia. After admission, patients underwent cardiopulmonary exercises for 1–6 months to improve exercise tolerance.

General anesthesia was selected. Etomidate combined with opioids was used during anesthesia induction to minimize effects on myocardial contractility and SVR, thereby reducing the risk of myocardial depression, decreased venous return, or exacerbation of hypoxia or hypercapni (26, 27). BIS monitoring was used to guide the depth of anesthesia to avoid circulatory inhibition caused by excessive depth of anesthesia. Maintaining an adequate MAP during the operation is crucial to ensure good spinal cord perfusion pressure (28). However, kyphosis correction often involves significant blood loss, and controlled hypotension techniques are employed to reduce intraoperative bleeding (29, 30). Consequently, somatosensory evoked potentials (SSEP) and myogenic motor evoked potentials (MMEP) should be monitored during the operation to ensure that the surgery or hypotension does not compromise spinal cord function (31, 32). Additionally, intraoperative monitoring of regional cerebral oxygen saturation (rSO2) is also beneficial to the management of hemodynamic parameters, early detection of hypoxia in the brain, spinal cord, kidney and other important organs, and formulation of organ protection strategies (33). Target-controlled infusion total intravenous anesthesia (TIVA) was used to maintain anesthesia, providing good controllability, comfort, and safety for intraoperative awakening. It is also essential to monitor blood gas, body temperature, and ventilation to avoid intraoperative arousal delay or difficulty caused by hypothermia, hypoxia, hypercapnia, acidosis, low hemoglobin (Hb), or hypovolemic blood volume.

In patients with kyphosis, thoracic deformities decrease lung compliance, lung volume, total lung capacity, vital capacity, and functional residual capacity (34–36). Impaired lung function can cause exercise intolerance (37, 38). A lung protective ventilation strategy is essential to manage increased airway pressure caused by thoracic and abdominal compression, impaired respiratory function, and prolonged operation time. Low tidal volumes can reduce the incidence of ventilator-associated lung injury (VALI) (39). However, decreased alveolar gas content can lead to alveolar collapse and ventilator-associated pneumonia. Appropriate PEEP and pulmonary resuscitation techniques can prevent atelectasis and alveolar collapse (40).

The PICCO system was used to evaluate hemodynamic parameters, such as continuous and dynamic cardiac index, extravascular lung water index, end-diastolic volume index, and systemic vascular resistance index during and after operation. It is increasingly used in critical operations and intensive care units (41), providing substantial help in guiding perioperative goal-oriented fluid therapy and follow-up treatment (42). Vasoactive drugs such as epinephrine and norepinephrine may be used to maintain or restore normal physiological function. Intraoperative autologous blood transfusion reduces the demand for allogeneic blood, lowers the incidence of transfusion reactions, and helps maintain hemodynamic stability (43). All operations were guided by target-directed fluid therapy under continuous monitoring and volume response evaluation of the lung protective ventilation strategy. The internal environment was corrected through intermittent arterial blood gas analysis (44). The intraoperative ratio of crystalloid to colloid infusion was 2:1, reducing the impact of large volumes of crystalloids on alveolar wall diffusion function and decreasing the incidence of pulmonary edema (45).

The key points of postoperative management include multi-mode pain management, early mobilization through rehabilitation exercises, and a nutritional support program (46). Both NSAIDs and selective COX 2 inhibitors, either alone or in combination, are effective in reducing postoperative pain (47). Additionally, IV-PCA can eliminate the gap between pain and analgesic administration, leading to better recovery and fewer side effects. This method is widely accepted for managing mild to severe postoperative pain (48). All patients need to perform long-term respiratory function exercises to adapt to postoperative respiratory changes. Early rehabilitation and physical exercise are essential for recovery.

## Data Availability

The original contributions presented in the study are included in the article/supplementary material, further inquiries can be directed to the corresponding author.
